# An Overview of the Epidemiologic, Diagnostic and Treatment Approaches of COVID-19: What do We Know?

**DOI:** 10.3389/phrs.2021.1604061

**Published:** 2021-06-17

**Authors:** Hanieh Beyrampour-Basmenj, Morteza Milani, Abbas Ebrahimi-Kalan, Ziyad Ben Taleb, Kenneth D Ward, Ghader Dargahi Abbasabad, Zeynab Aliyari-serej, Mohammad Ebrahimi Kalan

**Affiliations:** ^1^ Tabriz University of Medical Sciences, Tabriz, Iran; ^2^ University of Texas at Arlington, Arlington, VA, United States; ^3^ University of Memphis, Memphis, TN, United States; ^4^ Florida International University, Miami, FL, United States

**Keywords:** corona disease 2019, SARS-CoV-2, COVID-19, epidemiology, diagnosis, treatment

## Abstract

**Background:** In late December 2019, a new infectious respiratory disease (COVID-19) was reported in a number of patients with a history of exposure to the Huanan seafood market in China. The World Health Organization officially announced the COVID-19 pandemic on March 11, 2020. Here, we provided an overview of the epidemiologic, diagnostic and treatment approaches associated with COVID-19.

**Methods:** We reviewed the publications indexed in major biomedical databases by December 20, 2020 or earlier (updated on May 16, 2021). Search keywords included a combination of: COVID-19, Coronavirus disease 2019, SARS-CoV-2, Epidemiology, Prevention, Diagnosis, Vaccine, and Treatment. We also used available information about COVID-19 from valid sources such as WHO.

**Results and Conclusion:** At the time of writing this review, while most of the countries authorized COVID-19 vaccines for emergency use starting December 8, 2020, there is no a definite cure for it. This review synthesizes current knowledge of virology, epidemiology, clinical symptoms, diagnostic approaches, common treatment strategies, novel potential therapeutic options for control and prevention of COVID-19 infection, available vaccines, public health and clinical implications.

## Background

**FIGURE 1:**
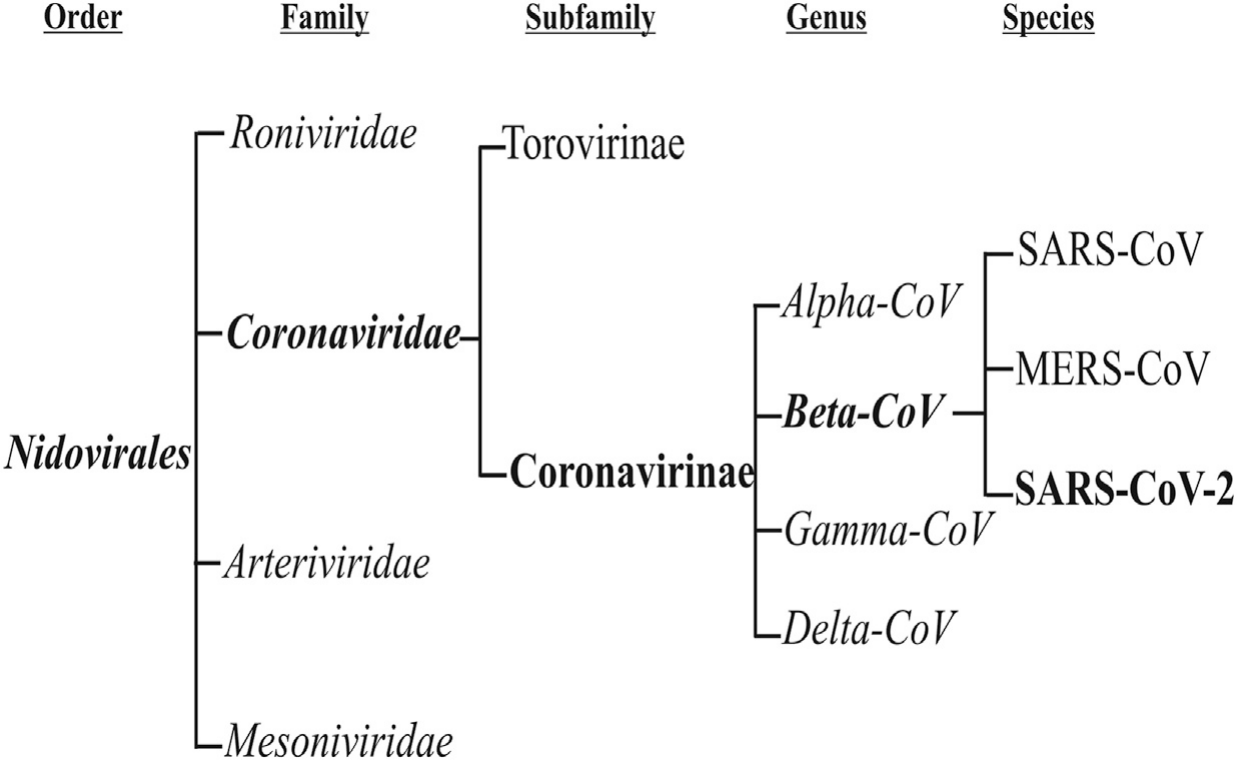
The taxonomy of the order Nidovirales.

Coronaviruses are a group of viruses that can cause respiratory infection in humans [1]. These viruses were discovered in 1966 by Tyrell and Bynoe, who cultured the viruses from common cold patients [2]. Because of their morphology as sphere-shaped viruses with a core-shell and surface projections similar to a corona (meaning “crown” in Latin), they are named coronaviruses [3, 4]. These viruses are members of the subfamily Coronavirinae in the family Coronaviridae and the order Nidovirales. This subfamily includes four groups: Alphacoronavirus, Betacoronavirus, Gammacoronavirus, and Deltacoronavirus (Figure 1). The Alpha and Betacoronaviruses infect just mammals. Although Gamma and Deltacoronaviruses infect birds, some also may infect mammals [5]. Alpha and Betacoronoviruses are usually associated with human respiratory disease and gastroenteritis disorders in animals. Middle East respiratory syndrome coronavirus (MERS-CoV) and Severe acute respiratory syndrome coronavirus (SARS-CoV) are the most pathogenic viruses which cause the intense respiratory syndrome. The other four human coronaviruses cause mostly mild to severe respiratory illnesses, (i.e. HCoV-229E, HCoV- NL63, HKU1, and HCoV- OC43) [1, 6, 7].

On New Year’s Eve 2019, a new, human-infecting coronavirus was reported in some patients with a history of contact with the Huanan seafood market, in Hubei Province, China [8]. This outbreak spurred Chinese scientists to sequence the genome of this virus immediately and raised concern about the link between this virus and wildlife, most likely from bats or pangolins, although a slew of genetic analyses has yet to find definite proof [8, 9]. Using next-generation sequencing, they reported that this virus belongs to B lineage of the Betacoronaviruses and is associated with the SARS-CoV [8, 10]. At first, the virus taxonomy was called 2019-nCoV, but, on February 11, 2020, the International Committee on Taxonomy of Viruses officially renamed the novel coronavirus responsible for the current pandemic of coronavirus disease (aka., COVID‐19), severe acute respiratory syndrome coronavirus 2 (SARS‐CoV‐2) [10]. Therefore, the virus is referred to as SARS-CoV-2, and the associated disease as COVID-19. At the time of last revision for this article (May 16, 2021), more than 162 million cases were reported globally with over 3.3 million deaths from 223 countries, areas or territories, mostly from the United States, India, Brazil, France, Turkey, Russia, United Kingdom, and Italy [11, 12]. As of May 12, 2021, a total of 1,264, 164, 553 vaccine doses have been administered, mostly in developed countries [11, 12]. Given the novelty and the lack of knowledge regarding the current pandemic of COVID-19, here we reviewed the existing evidence on epidemiologic, diagnostic, and treatment approaches of COVID-19.

## Virology

**FIGURE 2:**
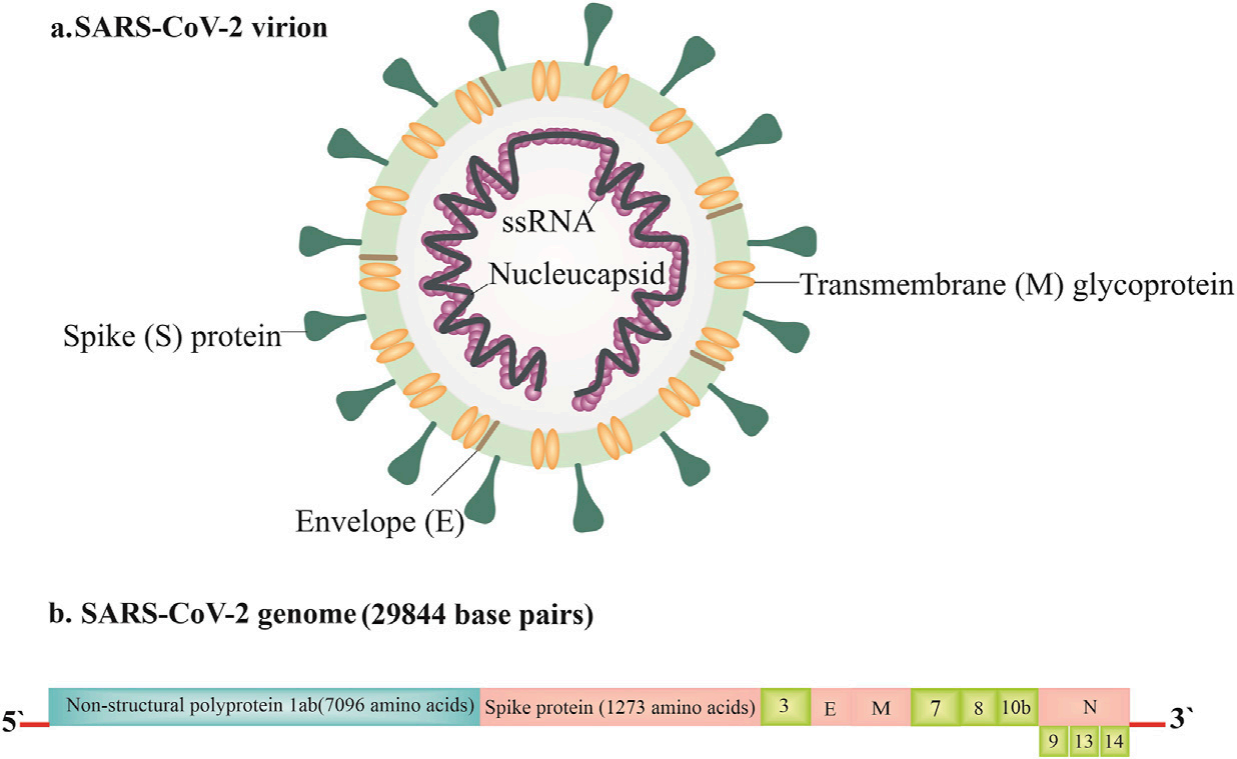
The severe acute respiratory syndrome coronavirus 2 (SARS-CoV-2) genome structure and virion [8].

The 2002–2004 SARS-CoV is a Betacoronavirus which first emerged in China and infected >8,000 people, leading to 774 deaths in 37 countries around the world [13, 14]. The MERS-CoV was known for the first time in Saudi Arabia in 2012 and resulted in 2,494 definite cases of infection and 858 deaths since September 2012 [15–17]. The receptor which SARS-CoV utilizes for contaminating type II pneumocytes and ciliated bronchial epithelial cells is angiotensin-converting enzyme 2 (ACE2) [18–20], while MERS- CoV employs dipeptidyl peptidase 4 [21] (DPP4) receptor and contaminates type II pneumocytes and unciliated bronchial epithelial cells [22–24]. SARS-CoV and MERS-CoV were spread to individuals from market civets and camels, respectively, and both of them are suspected of originating in bats [25–31]. Figure 2 displays the SARS-CoV-2 genome structure and virion.

SARS-CoV-2 is a single-stranded, sense positive RNA virus in length around 26–32 kb with a diameter of 60–140 nm [32]. The membrane of the virus comprises the transmembrane glycoprotein (M), spike glycoprotein (S), envelope protein (E), and a flexible nucleocapsid [33, 34]. Coronaviruses have a minimum of six open reading frames (ORFs). ORF1a/b is the first which contains around two-third of the genome and codes replica proteins. The 3′-end of the genome codes structural proteins with a set of additional proteins that are exclusive for each virus species. Coronavirus cell entrance occurs by binding the S protein to a certain receptor on the cell surface, subsequent fusion is facilitated by the target cell membrane, and finally, the virus nucleocapsid enters the cell for later replication [35]. Some studies indicate that COVID-19 uses ACE2 as its receptor-like SARS-CoV, suggesting that this virus can share a similar life cycle with SARS-CoV [4]. The clinical analysis showed that the S protein of SARS-CoV-2 binds 10- to 20- fold higher affinity than the S protein of SARS-CoV to ACE2. This high affinity may explain the ease by which SARS-CoV-2 can spread among the human populations [36]. Moreover, it has been confirmed that SARS-CoV-2 shares 96% nucleotide identity with the complete genome sequence of the bat coronavirus. However, attempts to recognize intermediate hosts await future research to elucidate [4]. The physicochemical features of SARS-CoV-2 have not been defined completely yet. It is reported that SARS-CoV-2 is sensitive to heat, lipid solvents like 70% ethanol, and UV radiation [37].

In a recently published study, genetic analysis was performed on 103 genomes of SARS-CoV-2 and the results indicated that SARS-CoV-2 developed into main types, L and S. These two types are defined by two single-nucleotide polymorphism (SNP) which demonstrated nearly complete linkage across SARS-CoV-2 strains. Also, the evidence proved that type L (70%) was more prevalent and aggressive than type S, but human interference probably shifted the abundance of L and S type after the outbreak of SARS-CoV-2 [38]. Nevertheless, as highlighted by authors of this study, it is pivotal to further investigate combine genomic and epidemiological data, and chart records of the clinical symptoms of patients infected by SARS-CoV-2.

## Epidemiology

Understanding of the epidemiology of COVID-19 is evolving since its first report to the Country Office of WHO in China on December 31, 2019 [39]. The exponentially increasing number of confirmed cases and deaths reported from China and beyond spurred WHO to declare a Public Health Emergency of International Concern on January 30, 2020, which was later upgraded to a declaration of COVID-19 pandemic on March 11, 2020 [40].

Symptoms of SARS-CoV-2 differ in some respects from other betacoronavirus, (e.g. SARS-CoV, MERS-CoV). For example, unlike SARS-CoV and MERS-CoV, SARS-CoV-2 spreads rapidly [41]. Surprisingly, this virus can infect and actively reproduce in the upper respiratory tract given that its close genetic relative, SARS-CoV, lacks this ability [42]. Moreover, whereas COVID-19 patients present intestinal symptoms like diarrhea only a low percentage of SARS-CoV or MERS-CoV patients exhibited these symptoms (Table 1) [43, 44]. However, a recent study in the New England Journal of Medicine [45], concluded that the stability of SARS-CoV-2 is comparable to that of SARS-CoV under clinical conditions. The authors suggest that dissimilarity in the epidemiologic features of these two viruses arise from other aspects such as high viral loads in the respiratory tract and the potential for asymptomatic individuals to shed and transmit the virus to other people. These features facilitate the transmission of the virus during daily activities and interactions between people. Given these characteristics, and to protect the population’s health and slow the rate of transmission of COVID-19, while waiting for vaccines to reach all over the globe, WHO and public health experts are urging several simple mitigation strategies, (e.g. face-covering masks, social distancing, and cleaning hands with an alcohol-based hand rub or washing them with soap and water) to combat COVID-19 pandemic [46–49].

**TABLE 1:** Comparison of the most common clinical symptoms of Common cold, Influenza, SARS, MERS, and COVID-19.

Disease	Clinical symptoms
Common cold	• sneeze, stuffy nose, runny noses
Influenza	• high fever, stuffy nose, muscle ache, dry cough, myalgia, and sore throat
SARS-CoV	• fever, chill, myalgia, headache, diarrhea, cough, dyspnea
MERS-CoV	• sore throat, dyspnea, myalgia
COVID-19	• fever, cough or not, muscle ache, headache, confusion, breathless, respiratory failure

Although understanding COVID-19 transmission risk is in its infancy, as the outbreak progressed, human-to-human spread was identified as the main mode of transmission [50]. The United States Centers for disease Control and Prevention (CDC) report shows that the major route of transmission of this virus is through respiratory droplets generated when an infected individual coughs or sneezes [51]. Additional evidence shows that close social interaction and touching the eyes, nose, or mouth with a contaminated hand, are sources of transmission [50, 52]. Whether transmission may happen via mother-infant or breast milk is not fully known yet [37]. For example, WHO scientific report (June 23rd, 2020) highlighted that the data are not enough to conclude vertical transmission of COVID-19 through breastfeeding [53]. Also, as of May 13, 2021, the United States Center for disease Control and Prevention (CDC) maintains that breast milk is not likely to spread the SARS-CoV-2 to babies [54]. A comprehensive review article published in the International Breastfeeding Journal on September 14, 2020, concluded that breastfeeding must be encouraged, mothers and newborns dyads should be cared for together, and skin-to-skin contact ensured throughout the COVID-19 pandemic [55]. Nevertheless, an earlier study from China concluded that vertical maternal-fetal transmission cannot be ruled out since three of 33 infants (9%; in their study sample) presented with early onset SARS-CoV-2 infection [56]. They suggested that it is critical to screen pregnant women and implement strict infection control procedures, quarantine mothers who are COVID19 positive, and closely monitor neonates at risk of COVID-19 [56]. Moreover, a recent case study concluded that transplacental transmission of SARS-CoV-2 infection is possible during the last weeks of pregnancy [57]. Although future longitudinal studies are warranted to better understand mother-to-child transmission, US CDC recommends sevral precautions while newborn is rooming-in with infected mother in the hospital [54]; they include wearing mask, washing hands with soap, keeping newborn more than 6 feet away from mother as much as possible, and using a physical barrier, (e.g. placing the newborn in an incubator).

As discussed earlier, the number of confirmed cases is increasing exponentially worldwide. As shown in Table 2, according to the WHO, as of May 16, 2021, the top 10 countries that are contributing to the ongoing global pandemic of COVID-19 reported case fatality rate (CFR) between 0.87% in Turkey to 2.99% in Italy, with global CFR of 2.07% (Table 2) [12, 40]. In comparison, the 2002–2004 outbreak of SARS had a CFR of around 10%, while MERS killed 34% of infected people between 2012 and 2019 [58].

**TABLE 2:** Case fatality rate (CFR) of COVID-19 reported to WHO by May 16, 2021 [11, 12].

	Cases	Deaths	CFR (%)
Global	162,177,376	3,364,178	2.07
United States	32,574,504	579,664	1.78
India	24,684,077	270,284	1.09
Brazil	15,519,525	432,628	2.79
France	5,769,839	106,778	1.85
Turkey	5,106,862	44,537	0.87
Russian federation	4,940,245	115,871	2.35
United Kingdom	4,448,855	127,675	2.87
Italy	4,153,374	124,063	2.99
Spain	3,598,452	79,281	2.20
Germany	3,593,434	86,096	2.40

Note: The date of situation reports for some countries may differ due to the late update. CFR, case Fatality Rate.

## Clinical Manifestation

Appropriate case definition is critical in recognizing and responding to infectious outbreaks, as well as for clinical diagnosis and public health surveillance [59]. According to WHO [60], the case definitions of COVID-19 are as follow:

1) “A suspected case is: A. a patient with acute respiratory illness (that is, fever and at least one sign of respiratory disease, for example, cough or shortness of breath) AND with no other etiology that fully explains the clinical presentation AND a history of travel to or residence in an area that has reported local transmission of COVID-19 during the 14 days prior to symptom onset.” Or “B. a patient with any acute respiratory illness AND who has been a contact of a confirmed or probable case of COVID-19 during the 14 days prior to the onset of symptoms (see the definition of contact below).” Or “C. a patient with a severe acute respiratory infection (that is, fever and at least one sign or symptom of respiratory disease, for example, cough or shortness breath) AND who requires hospitalization AND who has no other etiology that fully explains the clinical presentation.”
2) “A probable case is a suspected case for whom the report from laboratory testing for the COVID-19 virus is inconclusive.”
3) “A confirmed case is a person with laboratory confirmation of infection with the COVID-19 virus, irrespective of clinical signs and symptoms.”

According to the existing evidence, the incubation period for COVID-19 varies from 2 to 14 days with a median of ∼5 days, which is similar to SARS [37, 61–65]. The most important clinical symptoms of the disease are fatigue, fever, and dry cough. Regardless of the atypical symptoms that were reported, fever was cited as the typical sign of COVID-19 infection (Table2) [66]. The disease severity ranges from mild self-restricting flu-like ailment to fulfillment pneumonia, respiratory failure, and death.

As reported by WHO [12], 80% of COVID-19 infections are mild or asymptomatic, 15% are severe, requiring oxygen, and 5% are critical infections, requiring ventilation. Risk of acquiring virus and severity of COVID-19 differs by sex, age, and other factors such as severe obesity [67, 68]. A study based on publicly released data from 1,212 confirmed patients infected with COVID-19 in Henan of China, showed that a majority of infected cases were among males (55% vs. 45%) [69]. The results highlighted two probable factors that lead to this gender difference including 1) The high expression and distribution of COVID-19 receptor (ACE2) in male patients and 2) The tendency for males to engage in wider social activities than females [69]. Additionally, the reduced vulnerability of women to COVID-19 infections may be related to the protection from sex hormones and X chromosome, which have a key function in immunity [70]. Nevertheless, to explore more precise gender differences of COVID-19, future studies are warranted. A recent retrospective cohort study of 6,916 patients with COVID-19 in the US, found severe obesity as a profound risk for death from COVID-19, particularly in male patients and younger populations [67]. Severe obesity is an intractable problem in the United States and many western nations and little likely can be done in the short-term to reduce its effects on COVID-19 progression except to recognize the increased risk of obesity and target obese patients early for stepped up care.

Presentation of COVID-19 in children presents somewhat differently from adults. Although earlier in the pandemic it was recognized that children have milder symptoms (compared to adults) and are less likely to be hospitalized [71], several studies reported a multisystem inflammatory syndrome in children (MIS-C) associated with COVID-19 [72–74]. Available data by the American Academy of Pediatrics shows that, as of May 6, in 46 states of United States, over 3.85 million children have tested positive for COVID-19 since the onset of the pandemic, and children represented 14.0% of all cases in the Unites States by May 6, 2021 [75]. Although children were 0%–0.4% of all COVID-19 deaths, and 19 states reported zero child deaths, the United States CDC’s report highlights that children are at risk for severe COVID-19 and public health authorities and clinicians should continue to track pediatric COVID-19 infection [76].

As shown in Table 3, three major forms of the clinical courses of COVID-19 have been recognized by the Chinese CDC:

• Mild form: patients have asymptomatic upper respiratory tract infection (URI) with minor pneumonia, this usually occurred in approximately 80% of COVID-19 patients [77, 78].
• Severe form, (i.e. severe pneumonia): dyspnea, respiratory frequency (number of breaths human takes per minute) ≥ 30/min, blood oxygen saturation (SpO2) ≤ 93%, PaO2/FiO2 ratio or P/F [the ratio between the blood pressure of the oxygen (*partial pressure of oxygen*, PaO2) and the percentage of oxygen supplied (*fraction of inspired oxygen*, FiO2)] < 300, and/or lung infiltrates >50% within 24–48 h; this usually occurred in 14% of COVID-19 patients [77, 78].
• Critical form: cases display the progression of the disease rapidly with organ failure. Including respiratory failure that wants mechanical ventilation. Cases with acute respiratory distress syndrome (ARDS) characterized by refractory hypoxemia, septic shock as well as severe pulmonary infection; this manifests in about 5% of COVID-19 patients [77, 78].

**TABLE 3:** Major forms of the clinical severity of COVID-19 infection [77].

Types	Common symptoms
Mild	• non-pneumonia and mild pneumonia; seen in 81% of cases
Severe	• dyspnea
	• respiratory frequency ≥30/min
	• blood oxygen saturation (spo2) ≤ 93%
	• blood gas: pao2 < 60 mm hg, paco2 > 50 mmhg
	• prolonged prothrombin time and high level of D-dimer
	• rhabdomyolysis
	• elevated level of liver enzyme, (e.g. alanine aminotransferase (ALT))
Critical	• respiratory failure that wants mechanical ventilation
	• septic shock
	• severe pulmonary infection
	• multiple organ dysfunction (MOD)

## Strategies in COVID-19 Diagnosis

The United States CDC recommends the collection of specimens for COVID-19 examination from the respiratory tract [70]. Other specimens such as urine or stool can also be collected for diagnostic purposes. Following collection, specimens are tested in the form of reverse transcription-polymerase chain reaction (RT-PCR), which can determine results in 4–6 h [79, 80].

The WHO released a rapid advice guide on using chest imaging in COVID-19 patients on June 11, 2020 that highlights several points:

• “No study evaluated the diagnostic accuracy of chest imaging in asymptomatic patients possibly infected with SARS-CoV-2.”
• “In symptomatic patients in high COVID-19 prevalence cohorts, chest computed tomography (CT) appears to be associated with high sensitivity but low specificity, resulting in weak positive likelihood ratios and stronger negative likelihood ratios. This indicates that in these settings, negative imaging findings might be useful for ruling out COVID-19, but positive imaging findings are not useful for ruling in COVID-19.”
• “Evidence on the diagnostic accuracy of chest x-ray (CXR) was very limited but suggests lower sensitivity and possibly higher specificity than chest CT for diagnosing COVID-19.”
• “No study evaluated chest imaging in COVID-19 patients to inform decisions regarding discharge.”

Health providers collect serum samples for COVID-19 that may show virus spreading from the infected lung into the blood circulation, as formerly observed in SARS patients [81]. However, in the first infected case in the United States, it was reported that the stool and respiratory specimens were positive by RT-PCR for COVID-19, while the serum examination was negative [82]. Notably, in some patients, the ground-glass opacity (GGO) variations on CT scan revealed earlier than the positive for RT-PCR test [83, 84]. Thus, repeating sputum or nasopharyngeal swab test are suggested in suspected cases with an initially negative lab result taken from these samples [82, 85–87].

### Laboratory Examinations for COVID-19 Infection

In the disease initial phase, the white blood cell (WBC) count in COVID-19 patients can vary [88]. For example, leukopenia, leukocytosis, lymphopenia, and increased aminotransferase levels have been reported previously, although lymphopenia (lymphocyte count of less than 1.5 × 10^9^/L) seems most common [88–93]. It has been proposed that in disease early stage, the total amount of leukocytes is reduced or normal, monocytes amount is normal, and lymphocyte amount is decreased in peripheral blood [94]. Also, prolonged activated thromboplastin time (aPTT), increased muscle enzyme level and C-reactive protein (CRP) have been shown [66]. When the lymphocyte absolute value is less than 0/8 × 109/L, or the amounts of CD8+T and CD4^+^ cells are meaningfully reduced, high attention must be done that mostly suggested re-checking these indicators after 3 days [66]. Some investigations propose that a considerable reduction in the amount of lymphocytes shows that coronavirus targets immune cells and hinders the usual cellular immune function. Impairment in T lymphocytes can be a significant factor that leads to exacerbations of disease [70].

There are additional laboratory analyzes for the COVID-19 infection, such as renal and liver function test, blood gas examination with the increased level of lactic acid used for screening the cases with high-risk of oxygenation disorder, erythrocyte sedimentation rate (ESR), myocardial enzyme, myoglobin, Procalcitonin (PCT) for distinguishing bacterial infection in the lung, coagulation image, D-dimer, inflammatory factors, urine routine test, and anti-acid staining [66].

Bronchoalveolar lavage (BAL) is not recommended for the majority of patients because it increases the risk of infection. BAL can be considered, however, for cases that have noticeable signs of refractory massive atelectasis and airway obstruction that could not be returned by conservative treatments [37].

### Some Alternative Diagnostic Methods

COVID-19 diagnostic tests are widely available in a variety of types; however, most countries are still struggling with the management of pandemic [95]. Several novel techniques, including next generation sequencing, loop mediated isothermal amplification, and lateral flow tests are in different stages of validation [96, 97]. Each has benefits and drawbacks, so the best option depends on the intended use. Lateral flow tests designed to detect only infectious cases, can be easily scaled up for decentralized testing, inexpensive, and do not need laboratories [98]. On the other hand, they are less sensitive than RT-PCR, and very susceptible to poor sampling [98, 99]. When it comes to the affordability, accuracy, accessibility, and rapidness of results, no test is flawless. If the COVID-19 pandemic progresses, diagnostic methods may need to be optimized. Risk reduction strategies will aid in ensuring that research outcomes are reliable enough to provide adequate treatment at the patient level, as well as providing sound evidence to inform public health action to interrupt the transmission of the virus [95].

### Imaging Examination (CT Imaging)

**FIGURE 3:**
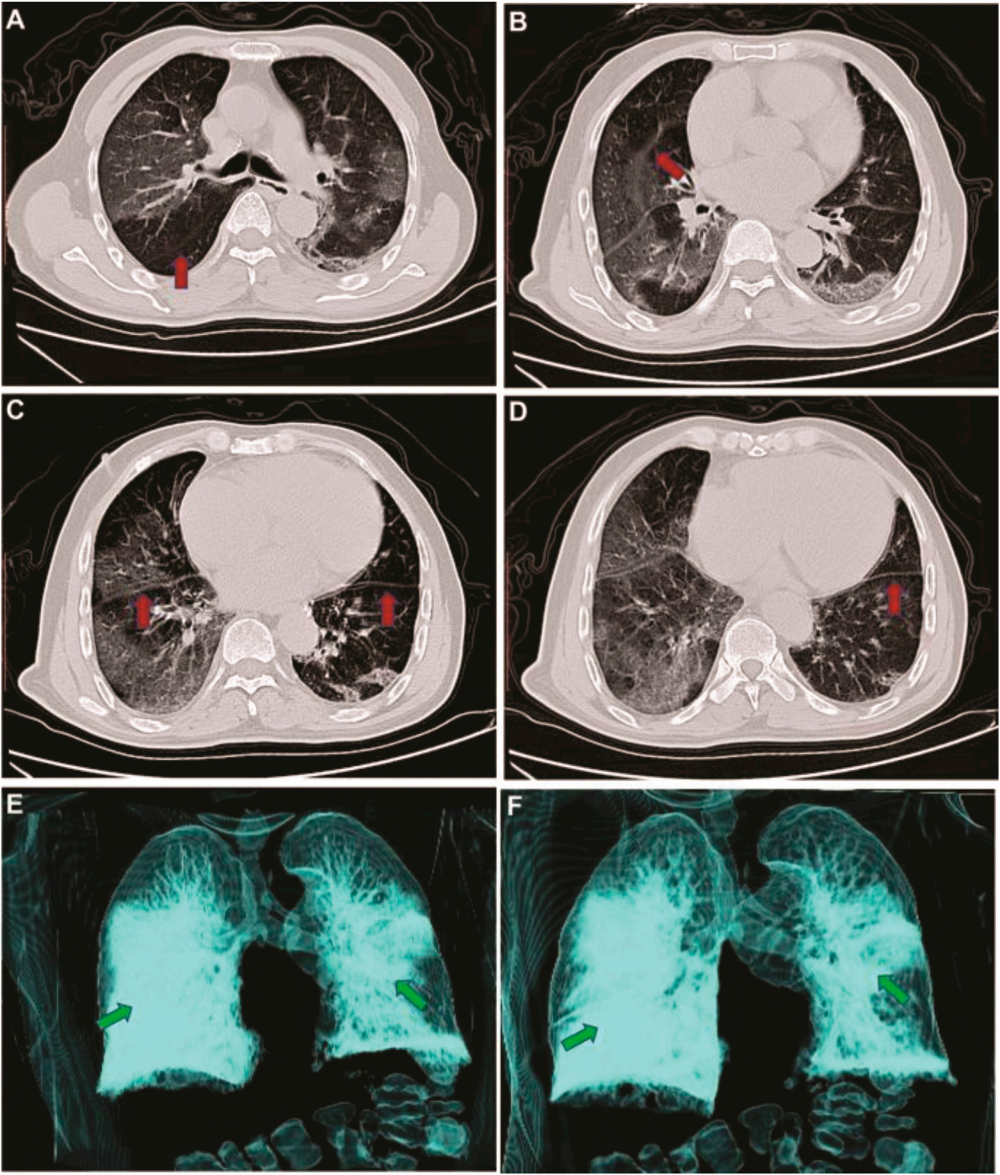
CT image changes of an infected patient with COVID-19. Panel A, B, and C indicate CT images on day 5 after symptoms onset, and D, E, and F represent on day 20 after symptoms onset. Adapted from Zhang, W. Imaging changes in severe COVID-19 pneumonia. Intensive Care Med [212].

Radiological analyses play a vital role in the diagnosis and control of COVID-19. As shown in Figure 3, the imaging results change with the patient’s age, disease stage, immunity status, drug interventions. The imaging findings can show the distribution, quantity, shape, and density of the lesions [66].

### Stage of the Disease Based on CT Image

As shown in Table 4, the CT scan reveals 5 phases based on onset time and the reaction of body to the infection [66], including:

1) Ultra-early, which typically indicates the cases without symptoms, negative laboratory examination but positive throat swab for COVID-19.
2) Early, this phase refers to the 1–3 days after signs appear. Some of the pathological symptoms in this phase are alveolar septal capillary congestion, fluid exudation in alveolar cavity, and interlobular interstitial edema.
3) Rapid progression, this phase refers to 3–7 days after clinical signs appear, the pathological manifestation in this phase is the accumulation of cell-rich exudates in the alveolar cavity, vascular expansion, and exudation in the interstitium, which result in aggravation of alveolar and Interstitial edema.
4) Consolidation, this phase refers to 7–14 days after symptoms appear. The key characteristics in this phase are the alveolar cavity fibrous exudation, and the capillary congestion disappearance in the alveolar wall.
5) Dissipation, this phase refers to the time around 2 and 3 weeks after the beginning of signs, in which the range of lesions decreases.

**TABLE 4:** Stage of the disease according to CT imaging results.

Stage	CT characteristics
Ultra-early	• single or scattered focal GGO
	• nodules in central lobule
	• patchy consolidation
	• intra-bronchial air-bronchogram
Early	• single or multiple scattered patchy or agglomerated GGO
Rapid progression	• large-scale light consolidation with air-bronchogram
Consolidation	• multiple patchy consolidations in smaller density
Dissipation	• patchy consolidation or strip-like opacity

While chest CT scan results are not specific for COVID-19 diagnosis, CT results have been suggested as the main confirmation of medical diagnosis of this infection [100]. A study that initially performed an RT-PCR test, and in the case of a negative result repeated testing at intervals of 1 day or more, found that chest CT scans had greater sensitivity for diagnosis compared with initial RT-PCR from respiratory specimens ((98% vs. 71%, respectively; *p* < 0.001) [101]. To date, chest CT scan and RT-PCR remain the most common and available first clinical options in diagnosing COVID-19 infection worldwide [100].

## Treatment Approaches for COVID-19 Infection

In the current situation, controlling the infection is the best way to tackle the pandemic of SARS-CoV-2 [102]. Some non-pharmaceutical strategies, including early diagnoses, isolation, and supportive treatments and protective measures, (e.g. wearing masks, improving personal hygiene, and keeping rooms ventilated) can efficiently break the SARS-CoV-2 transmission chain [103].

Overall, treatment approaches are classified into three stages related to the severity of the disease:

1) Mild phases of the disease: symptomatic support treatment [104].
2) Severe phases: oxygen inhalation is required for patients with emergency symptoms and SpO2 < 93%. Dynamic assessment of the patient’s oximeter and regularly monitor patient’s symptoms by laboratory examination or CT imaging [104, 105].
3) Critical phases: protective mechanical ventilation after tracheal intubation is necessary, and prone position ventilation followed if P/F ratio does not get better, and ultimately extracorporeal membrane oxygenation (ECMO) should be applied [104].

Analysis of the SARS-CoV-2 RNA genome indicated that this virus has differences in sequence. These differences are typically single nucleotide variations. The preliminary data from samples suggest that SARS-CoV-2 is actively evolving through various mutations which can lead to a major challenge in developing effective drugs and vaccines [106].

At the time of writing this review, no definite cure has been provided for COVID-19. Some drugs, however, are prescribed by health providers, such as nucleoside analogs, lopinavir/ritonavir (LPV/r), umifenovir (Arbidol), neuraminidase inhibitors, remdesivir, DNA synthesis inhibitors (like Disoproxil, tenofovir, and Lamivudine), Hydroxychloroquine/Chloroquine (HCQ/CQ), and favipiravir [107, 108]. There are some positive reports that these antiviral drugs may have clinical efficacy against COVID-19 [109].

For patients with severe signs, antibiotics (Tazobactam sodium, Moxifloxacin, Piperacillin sodium, and Meropenem) and methylprednisolone (1–2 mg per kg weight per day) also have been utilized, according to the physicians’ decision [70]. In low immune function cases, like diabetics, older people, HIV infected persons, individuals with long-term use of immunosuppressive drugs, and pregnant women, early prescribing of antibiotics to inhibit infection may decrease morbidity and mortality [70].

Since the start of the COVID-19 pandemic, corticosteroid use has been the subject of debate.

Previously, it has been recommended that immune-modulating drugs such as corticosteroids be utilized in some special situations, including: in quickly deteriorating chest imaging and ARDS, in encephalitis, hemophagocytic, septic shock, and in apparent wheezing symptoms. Intravenous methylprednisolone (one to two mg/kg/day) usage is suggested for 3–5 days [37, 110].

However, according to the latest evidence corticosteroids do not appear to play a main role in treatment of ARDS [111, 112]. Despite improved cardiopulmonary physiology, routine use of methylprednisolone for persistent ARDS is not recommended [112].

According to a meta-analysis of COVID-19 patients, mortality was higher among patients receiving corticosteroids than among patients who were not receiving corticosteroids [113]. In contrast, a cohort study of 1444 COVID-19 patients found that treating with corticosteroids had no effect on hospital mortality [114]. As a result, corticosteroids seem to be a double-edged sword in the battle against COVID-19 and should be used cautiously, considering the risk–benefit ratio [115].

These recommendations are supported by WHO guidance suggesting that corticosteroid regimens should not be routinely used in the treatment of COVID-19 except in certain scenarios such as treatment of exacerbated asthma attacks, COPD, or septic shock [110, 116].

### An Overview of Selected Repurposed Drugs

Remdesivir, a nucleotide analog prodrug, was initially developed by an American biotechnology company Gilead Sciences against the Ebola virus. It has been shown to have potential as an anti-viral drug for SARS-CoV-2. Evidence from a small cohort showed clinical improvement in 68% of patients treated with remdesivir [117]. The United States NIH reported that patients in the remdesivir group showed a 31% faster recovery time compared to those in the placebo group [118]. So, remdesivir could have a considerable impact on healthcare capacity by reducing the length of time in hospital [119, 120].

Previous studies indicate the combination of lopinavir/ritonavir (HIV protease inhibitors) can be helpful for MERS-CoV and SARS-CoV infected cases [121, 122]. While LPV/r has been shown to be effective in patients infected with SARS-CoV [121, 123], but it showed less potent than other antiviral drugs such as remdesivir and Chloroquine [124]. In a study involving 199 COVID-19 patients, LPV/r failed to decrease mortality and viral load [125]. Therefore, it is challenging to determine if the LPV/r can play an effective role in dealing with COVID-19 [126].

Hydroxychloroquine/Chloroquine (HCQ/CQ) are anti-malarial drugs, but also have been suggested as promising agents against the COVID-19 [127]. HCQ/CQ showed *in vitro* activity for SARS-CoV-2 with an EC50 value of 23.90 and 6.14 μM [128, 129]. However, there are inadequate data to support the use of HCQ/CQ as therapeutic agents in COVID-19. The evidence demonstrated significant virologic clearance in the HCQ group and also the HCQ plus Azithromycin group showed considerably better virus clearance [130, 131]. A recent study on 96,000 patients indicated an increased incidence of ventricular arrhythmias in patients who treated with Hydroxychloroquine or Chloroquine [132]. Likewise, the United States Food and Drug Administration (FDA) has warned against using of HCQ/CQ as a therapeutic agent for COVID-19 because of the risk of Adverse Drug Reactions (ADR) [133]. Additionally, a multicenter, randomized clinical trial among 504 hospitalized patients with suspected or confirmed COVID-19 who were receiving either no supplemental oxygen or a maximum of 4 L per minute of supplemental oxygen, found that the use of Hydroxychloroquine, alone or with azithromycin, did not improve clinical status at 15 days as compared with standard care [134]. It is pivotal to know that Hydroxychloroquine has the potential for cardiac toxic effects and overall adverse consequences have been emphasized, especially in people with underlying coexisting conditions that increase the risk of severe COVID-19 [135].

Favipiravir is an anti-viral drug that inhibits RNA-dependent RNA polymerase (RdRp). It is used for treating Influenza [136, 137]. The *in vitro* activity of it was shown against SARS-CoV-2 [129]. Favipiravir has been proved for use in patients with COVID-19 in some countries such as Russia and China [138, 139]. However, favipiravir did not show a noteworthy benefit in infected cases in a trial in Japan [140].

### Convalescent Plasma Therapy

In CPT, plasma donated by people who have recovered from COVID-19, the donated plasma is transfused to patients who are ill, so their antibodies will help in overcoming the virus. Studies on patients with COVID-19 showed clinical improvements after CP therapy [141–144]. Recently it has been shown that transfusion of CP is safe and does not have adverse effects on COVID-19 patients also reported a reduction of mortality [145, 146]. The main problem for large-scale plasma therapy is finding donors with high levels of antibody titer (>1:640) [142]. Evidence indicated that neutralizing antibodies reduced quickly after four months of the recovery [147]. This transient immune response recommends that plasma from freshly recovered cases is more efficient for CPT. Still, more investigation is required to know about the concentration and type of neutralizing antibodies in order to protect against the virus [148, 149].

### Monoclonal Antibodies for Treating SARS-CoV-2 Infection

Monoclonal antibodies (mAbs) can be obtained from laboratory preparation or by COVID-19 patients. Numerous mAbs are approved by the FDA to treat different disorders such as cancer [148]. Moreover, few monoclonal antibodies are utilized for treating SARS-CoV-2 like tocilizumab [150–152], bevacizumab [153, 154], etc [148].

ACE2, Spike proteins, and their interactions are the key targets for developing novel therapeutic monoclonal antibodies [155–157]. Different neutralizing mAbs are tested against SARS-CoV including m396, CR3022, CR3014, 80R, B1, 68, 201, 5E9, and 1F8, these antibodies could have a critical therapeutic function in SARS-CoV-2 infections [155, 158, 159]. A summary of some of the therapeutic agents that have been applied for COVID-19 treatment was demonstrated in Table 5.

**TABLE 5:** Summary of potential therapeutic drugs for COVID-19.

Intervention	Type	Mechanisms	References
Remdesivir	Antiviral	Remdesivir interferes with virus RNA polymerases to inhibit virus	[120, 129]
Lopinavir/ritonavir	Antiviral	Inhibits 3CL protease activity, blockage of protein processing	[123]
Favipiravir	Antiviral	Inhibits viral RNA polymerase, thus interfering with viral replication	[137]
Hydroxychloroquine/Chloroquine	Antiviral/Antimalaria	Endosomal acidification fusion inhibitor anti-inflammatory activity	[129]
Convalescent plasma	Antiviral	Convalescent plasma from cured patients provides a protective antibody against SARS-CoV-2	[144]
Tocilizumab	mAb	Interleukin-6 inhibitor, humanized mAb targeting IL-6, immunosuppressive, blockage of a cytokine storm	[151, 152]
Bevacizumab	mAb	Humanized mAb targeting VEGF	[154]

### COVID-19 Vaccines

Vaccination is one of the most important strategies and among the safest medical products to prevent infectious diseases from spreading nationally and internationally. The exponentially increased number of patients in the first days of this pandemic triggered intense global research and development activities to use several technologies including protein subunit, viral-vectored, nucleic acid (DNA, RNA), live attenuated and inactivated vaccines with some entering clinical trials to develop a safe and efficient vaccine against this novel disease [160]. According to the up-to-date list by the WHO [161], as of May 14, 2021, more than 180 COVID-19 vaccines were being in the preclinical development and 100 were in clinical development across the globe.

Companies such as BioNTech and Pfizer developed mRNA vaccine BNT162 against COVID-19 [162]. They found the efficacy rate of the vaccine nearly 95%. It has been revealed that two weeks after the first dose, the vaccine started protecting, and the second dose, three weeks later, enhanced the immune response of the volunteers [163]. On December 11, New York-based Pfizer and the German company BioNTech got the first emergency use authorization from the FDA for COVID-19 vaccine [163]. Despite its early vaccination process, on December 19, 2020, the United States CDC reported some people have experienced severe allergic reactions—also known as anaphylaxis—after receiving a COVID-19 vaccine [164]. CDC recommends that “People with a history of severe allergic reactions should be monitored for 30 min after getting the vaccine, while all other people should be monitored for 15 min after getting the vaccine.” [164].

Moderna, an American biotechnology company based in Cambridge, Massachusetts developed the mRNA-1273 vaccine against SARS-CoV-2, which produced by mRNA technology. For mRNA-1273 vaccine, a phase 1 study of eight participants reported in May 2020, showed a higher immunocompatibility similar to those seen in patients formerly infected with COVID-19 [165–170]. On June 11, Moderna announced that the cohort of healthy younger adults ages 18–55 (*n* = 300) and the sentinel group of older adults ages 55 years and older (*n* = 300) in the phase 2 study of mRNA-1273 was completed [171]. Moderna has started its phase 3 trial with 30,000 volunteers in conjunction with the Unites States National Institute of Allergy and Infectious Diseases (NIAID) Moderna has declared that the vaccine had an efficacy rate of 94.1% [163, 165–170]. On December 18, 2020, the United States FDA authorized Moderna Vaccine for emergency use in the United States a week after authorizing BNT162 [172]. In addition, the United States-based company Novavax is developing the recombinant vaccine NVX-CoV2373 as a stable, perfusion protein advanced by the nanoparticle technology [173]. They put the genetic code for the spike protein into the genome of yeast or bacterium, so it can produce a large amount of protein [174].

The ChAdOx1 nCoV-19 vaccine that was developed by the University of Oxford containing the full-length sequence of COVID-19 spike protein [175–177] with acceptable safety profile [178], gained authorization for emergency use in the United Kingdom, and 90 year-old Margaret Keenan was the first person to get vaccinated on December 8 [179].

INOVIO has developed the DNA plasmid vaccine as an INO-4800. The company has established such platforms for MERS and SARS viruses [180]. This approach needs a delivery system through electroporation, which will increase the cost of the vaccine [180]. A lately study in *The Lancet* announced a new effective vaccine for SARS-CoV-2 named PittCoVacc, established by University of Pittsburgh [181]. PittCoVacc utilizes S-protein fragments of SARS-CoV-2 for stimulating the production of antibodies. PittCoVacc is delivered with a method identified as microneedle array, a fingertip-sized patch of 400 small needles. This approach can cause more immune responses than conventional subcutaneous injection and has been proved to be sufficiently safe [182].

On July 11, 2020, Russian President Vladimir Putin announced that Russia had become the first country to approve a COVID-19 vaccine (called “Sputnik V”) [183]. Although some researchers warned about Russian’s rushed decision [183, 184], a most recent interim analysis of the phase 3 trial of Gam-COVID-Vac among adults aged 18 years or older that was published in *The Lancet* demonstrated 91·6% efficacy against COVID-19 [185]. A summary of developed vaccines is presented in Table 6. A most recent data released on May 15 by Italian scientists shows that COVID-19 infections in adults fell by 80% five weeks after receiving a first dose of Pfizer, Modernaor AstraZeneca vaccines [186]. This might be higher after second dose of the vaccin and awaits further studies to explore.

**TABLE 6:** Summary of the candidate COVID-19 vaccines in clinical/post-clinical evaluation.

Name of vaccine	Mechanisms	Location	References
mRNA-1273	Lipid nanoparticle encapsulated mRNA (RNA) that can code S protein of SARS-CoV-2	United States	[167]
ChAdOx1	Attenuated adenovirus vector able of generating the S protein of SARS-CoV-2	United Kingdom	[176]
BNT162	mRNA vaccine expressing codon-optimized undisclosed SARS-CoV-2 proteins	Germany	[162]
INO-4800	DNA plasmid vaccine	United States, South Korea	[180]
Ad5-nCoV	Adenovirus type 5 as vector for loading some gene fragments of SARS-CoV-2 in order to express S protein	China	[213]
PittCoVacc	Employs microneedle array for delivering pieces of S-protein into body	United States	[181]
NVX-CoV2373	A stable, perfusion protein established by nanoparticle technology	United States	[173]

Serological tests to detect specific antibodies for the SARS-CoV-2 are under development [187]. These tests can help identify individuals who have been infected and developed immunoglobulin M (IgM) and/or immunoglobulin MG (IgG) antibodies that may protect from potential future infection (post-infection immunity) [188, 189] as well as identify those who remain at risk through human leukocyte antigen A [HLA-A], -B, and -C genes [190].

Since IgM antibodies in infected patients may not develop early or at all, this type of antibody test cannot rule out SARS-CoV-2 in an individual. A recent clinical trial of 36 patients with COVID-19 infection and 42 patients with other viral respiratory infections which were published in the Nature Biotechnology reported that the clustered regularly interspaced short palindromic repeats (CRISPR)-based detector essay provides a visual and faster alternative to the United States CDC SARS-CoV-2 real-time RT–PCR assay, with 95% positive predictive agreement and 100% negative predictive agreement [191]. Even with these positive reports concerning antibodies, the majority of scientists believe that most of the antibody tests that were released to the market without appropriate scrutinize and reviews are not accurate enough to confirm whether an individual has been exposed to the virus [192]. Therefore, to understand the full effectiveness of these antibodies, for future large clinical trials and a comprehensive review with governmental agencies are warranted.

## Emerging SARS-CoV-2 Variants

On September 2020 Public Health authorities in South East region of England detected a new SARS-CoV-2 variant as a lineage B.1.1.7, aka Variant of Concern 202,012/01 (VOC-202012/01), 20I/501Y.V1, or more commonly known as the British variant or United Kingdom variant [193]. Seventeen mutations in the viral genome occurred in this variation, eight of which are localized in the spike (S) protein of the virus. Two significant characteristics of lineage B.1.1.7 are the N501Y mutation and the 69–70 deletion of the S protein. The 69–70 deletion reduce sensitivity to neutralization by convalescent serum samples [194]. The N501 is one of the six amino acids interacting with ACE-2 [195], and the tyrosine substitution could increase binding affinity to the ACE-2 [196]. These variations have led scientists to be worried about high contagiousness, rapid spread, and possibly augmented mortality.

Variant B.1.351 has appeared in South Africa over the last several months, and similar to the B.1.1.7 raised transmission rates and higher viral load following infection have been reported [197]. Mutations in the S protein are more extended than variant B.1.1.7. Three of these mutations are found in the RBD (E484K, K417N, N501Y). Since RBD is the dominant target for neutralizing antibodies, these mutations might affect the effectiveness of previously approved monoclonal and polyclonal antibodies elicited by vaccination or infection in neutralizing the virus [198, 199].

An additional independent lineage of SARS-CoV-2 (named P.1 variant or 20J/501Y.V3) was identified in Brazil. Variant P.1 is a branch of lineage B.1.1.28 that was initially reported by the Japanese National Institute of Infectious Diseases (NIID) in four travelers from Brazil. It contains three mutations in the spike protein receptor binding domain (E484K, N501Y and K417T) [200]. Existing evidence suggests that some mutations in the P.1 variant can affect its transmissibility and antigenic profile, that may affect the ability of antibodies produced by a previous infection or vaccination [201].

Globally, researchers constantly working hard to gain knowledge and richer understand the variations of virues alluded to above in order to provide information to public health authorities like WHO to prevent more infections and save lives.

## Mental Health in the Era of COVID-19

It is critical not to neglect the mental health issues associated with the COVID-19 outbreak. Patients, family members, and healthcare workers who treat viral outbreak patients are all at increased risk of developing mental health problems. For example, the 2002–2003 global SARS epidemic was labeled a “mental health catastrophe” [202]. Health care workers on the front lines of patient care during viral outbreaks experience a whole host of stressors, such as long shifts, difficulty balancing professional and family responsibilities, dealing with inadequate equipment and the fear for personal and family safety, and having to make ethically challenging decisions about the allocation of treatment resources. As a result, healthcare workers are at increased risk of experiencing psychological distress and post-traumatic stress disorder (PTSD) [203]. Additionally, COVID-19 patients, those patients who recovered but still have some symptoms like shortness of breath, high risk populations, (e.g. the elderly, people living with HIV, and those living or receiving care in crowded locations), and people with preexisting medical conditions such as psychiatric or substance use problems are at increased risk for adverse psychosocial consequences [204–206]. Therefore, providing psychosocial support and regularly monitoring mental health needs of those at higher risk necessitate activities that need to be integrated into the general pandemic healthcare system, especially for those nations that are hard hit by this novel disease.

### Conclusion

The new coronavirus, termed SARS-CoV-2, causes severe respiratory infection and has been spreading worldwide quickly. Yet, information regarding this novel virus remains inadequate. The main goal of worldwide efforts against COVID-19 in the absence of any approved pharmacological agents is to apply primary preventive measures e.g., face-covering with masks, social distancing, stay at home orders, closing places like theaters, isolation of suspected cases, using PPE in healthcare units, contact tracing and screening persons who might be infected in order to restrict the outbreaks [207, 208]. COVID-19 appears to transmit human-to-human via the same way as other common cold or influenza viruses. Chest CT scan findings and RT-PCR are considered first and available diagnostic approaches of COVID-19. Thin slice chest CT can be helpful in rapid detection, and disease control, playing an essential role in the initial diagnosis and monitor of COVID-19. In terms of laboratory examinations, it has been shown that the total number of lymphocytes in most cases is decreased. This result suggests that COVID-19 may mostly act on lymphocytes, particularly T lymphocytes. The low number of lymphocytes can be used as a reference index in the detection of COVID-19 infections in suspected cases. Therefore, early diagnosis and appropriate symptomatic treatments of critical cases have vital importance.

Lack of trained personnel, shortage in mechanical ventilators, failure to implement community preventive measures (especially in poor and under war countries), delay in lockdown, quick reopening in some countries, inadequate risk communication (“the exchange of real-time information, advice and opinions between experts and people facing threats to their health, economic or social well-being”) [209] and finally ICU occupancy are thought to be major reasons behind the high mortality associated with COVID-19 in most countries with the highest incident rate [210]. More importantly, it is crucial to adhere to global research ethical standards in developing vaccines, conducting clinical trials, and approving those vaccines to use in the general public. Therefore, to tackle these issues, countries (especially those with the highest number of COVID-19 deaths) need to follow WHO’s guidelines strictly to flatten the mortality curve and save lives. Additionally, WHO translates that knowledge into strategic action that can guide the efforts of all countries when developing context-specific national operational plans [211]. The rapid progress and authorizing three vaccines to be inoculated in the United States and United Kingdom have been almost revolutionary, spurred by the urgent need to blunt the pandemic that is wrecking hovoc on families, society, and economy worldwide.

Constant research is essential to recognize the source of the outbreak and finding the intermediate host for inhibiting future coronavirus outbreaks. Public health authorities must monitor the condition carefully and release surveillance data to better understand the behavior of this novel disease. Although much more remains to be done with many unanswered questions, proactive investment in public health infrastructure and resources is critical, including preventive education, (i.e. social distancing, washing hands, quarantine), vaccine and therapeutic development, providing sensitive and specific diagnostic tests, the availability of well-equipped hospitals for patients with critical conditions, and providing resources in the aftermath of the pandemic to optimize psychological adjustment in survivors.

## Abbreviations

ACE2, angiotensin-converting enzyme two; ADR, adverse drug reactions; aPTT, prolonged activated thromboplastin time; ARDS, acute respiratory distress syndrome; BAL, bronchoalveolar lavage; CDC, United States Centers for disease Control and Prevention; CFR, case fatality rate; COVID-19, coronavirus disease 2019; CPT, convalescent plasma therapy; CT, computed tomography; CRP, C-reactive protein; CXR, chest x-ray; DPP4, dipeptidyl peptidase four; ECMO, extracorporeal membrane oxygenation; ESR, erythrocyte sedimentation rate; GGO, ground-glass opacity; HCQ/CQ, hydroxychloroquine/Chloroquine; HLA-A, human leukocyte antigen A; LPV/r, lopinavir/ritonavir; mAbs, monoclonal antibodies; MERS-CoV, middle East respiratory syndrome coronavirus; MIS-C, multisystem inflammatory Syndrome in Children; NGS, next-generation sequence; NIAID, United States National Institute of Allergy and Infectious Diseases; ORFs, open reading frames; PaO2/FiO2 ratio or P/F, the ratio between the blood pressure of the oxygen (*partial pressure of oxygen*, PaO2)and the percentage of oxygen supplied (*fraction of inspired oxygen*, FiO2); PCT, procalcitonin; PTSD, post-traumatic stress disorder; RdRp, RNA-dependent RNA polymerase; RT-PCR, real-Time PCR; SARS-CoV, severe acute respiratory syndrome coronavirus; SNP, single-nucleotide polymorphism blood oxygen saturation upper respiratory tract infection World Health Organization.
